# Unprecedented early-summer heat stress and forecast of coral bleaching on the Great Barrier Reef, 2021-2022

**DOI:** 10.12688/f1000research.108724.1

**Published:** 2022-02-01

**Authors:** Blake L. Spady, William J. Skirving, Gang Liu, Jacqueline L. De La Cour, Cathy J. McDonald, Derek P. Manzello

**Affiliations:** 1 Center for Satellite Applications and Research, Satellite Oceanography and Climate Division, U.S. National Oceanic and Atmospheric Administration, College Park, Maryland, 20740, USA; 2 ReefSense Pty, Ltd., P.O. Box 343, Aitkenvale, Queensland, 4814, Australia; 3 Earth System Science Interdisciplinary Center, Cooperative Institute for Satellite Earth System Studies, University of Maryland, College Park, Maryland, 20740, USA

**Keywords:** Background temperature, Bleaching, Coral, Degree Heating Week, Great Barrier Reef, heat stress, La Niña, satellite monitoring

## Abstract

The Great Barrier Reef (GBR) is predicted to undergo its sixth mass coral bleaching event during the Southern Hemisphere summer of 2021-2022. Coral bleaching-level heat stress over the GBR is forecast to start earlier than any previous year in the satellite record (1985-present). The National Oceanic and Atmospheric Administration (NOAA) Coral Reef Watch (CRW) near real-time satellite-based heat stress products were used to investigate early-summer sea surface temperature (SST) and heat stress conditions on the GBR during late 2021. As of 14 December 2021, values of instantaneous heat stress (Coral Bleaching HotSpots) and accumulated heat stress over a 12-week running window (Degree Heating Weeks) on the GBR were unprecedented in the satellite record. Further, 89% of GBR satellite reef pixels for this date in 2021 had a positive seven-day SST trend of greater than 0.2 degrees Celsius/week. Background temperatures (the minimum temperature over the previous 29 days) were alarmingly high, with 87% of GBR reef pixels on 14 December 2021 being greater than the maximum SST over that same 29-day period for any year from 1985-2020. The GBR is starting the 2021-2022 summer season with more accumulated heat than ever before, which could have disastrous consequences for the health, recovery, and future of this critical reef system.

## Introduction

The Great Barrier Reef (GBR) has endured five mass coral bleaching events, three of which took place between 2016 and 2020. Coral bleaching occurs when stress disrupts the symbiosis between corals and their endosymbiotic algae (zooxanthellae), causing the corals to expel zooxanthellae; this can lead to coral mortality if stress is prolonged or severe ( Brown, 1997). Mass coral bleaching, which is bleaching at a scale of an entire reef system or geographic realm, has only been linked to the stress of excess sea surface temperatures (SST), and this is expected to happen when heat stress in a region exceeds a certain intensity or duration ( Glynn, 1984; Hoegh-Guldberg, 1999; Skirving *et al.*, 2019). The U.S. National Oceanic and Atmospheric Administration’s (NOAA) Coral Reef Watch (CRW) program has developed a number of satellite-based SST products that are used to monitor oceanic heat stress, including on coral reefs. The Degree Heating Week (DHW) is an accumulation of instantaneous heat stress (Coral Bleaching HotSpots, or just HotSpots) over a 12-week running window ( Skirving *et al.*, 2020). A DHW value of 4 degree Celsius-weeks (C-weeks) or greater is capable of causing sufficient stress for corals to bleach significantly ( Hughes *et al.*, 2018; Skirving *et al.*, 2019). In the early-summer months preceding the five documented mass bleaching events, heat stress on the GBR had never exceeded a DHW of 3 degree C-weeks prior to mid-January (with the earliest occurrence in 2002 on 12 January), with peak stress typically occurring between late February and early March. In late 2021, sections of the northern GBR reached a DHW ≥ 3 degree C-weeks by 13 December, and given the observed conditions at the time of writing (21 December 2021), roughly one third of the GBR is expected to exceed a DHW of 4 degree C-weeks by late January 2022, which is unprecedented.

In addition to the satellite-based measurements, NOAA CRW’s modelled Four-Month Coral Bleaching Heat Stress Outlook product, from as early as 2 November 2021, forecast sufficient heat stress to result in a potential sixth mass coral bleaching event on the GBR during the 2021-2022 summer season. The anticipated mass bleaching event was not only forecast to occur during atypical climatic conditions (i.e., this would be the first mass coral bleaching on the GBR during a La Niña), but was also forecast to be the earliest onset of a summer-time heat stress event for the GBR to date. During the eleven-year period covered by CRW’s Outlook product (2011-2021), the GBR has experienced three of its five known mass bleaching events, one of which (2020) occurred as a result of the most extensive heat stress event the region has suffered ( Hughes *et al.*, 2021). Here, we will describe the early-summer SST and heat stress conditions on the GBR in 2021 (November-December), modelled forecasts of heat stress, and how these compare to observations made in previous years, including the relationship between coral bleaching and the El Niño-Southern Oscillation (ENSO) state.

## Results and discussion

### Early-summer heat stress conditions of late 2021 compared to previous years

On 14 December 2021, 59% of the 5 km-resolution satellite-based reef pixels (0.05° × 0.05° satellite pixels that coincide with reefs) on the GBR had HotSpots greater than 0.5 °C, with 34% being greater than 1.0 °C. This is more extensive and severe than the HotSpots measured on 14 December during any year since 1985. The accumulation of HotSpots as DHWs for the same date reached a DHW > 2 degree C-weeks for 14% of GBR reef pixels. DHWs greater than 2 degree C-weeks have accounted for more than 0.1% of the GBR reef pixels by 14 December during only three other years (2008: 1.2%, 2010: 10.8%, 2018: 5.4%), none of which were as high as by 14 December 2021 (13.7%, Table 1). The seven-day SST trend as of 14 December 2021, indicated that SST had increased by at least 0.2 degrees C/week for 89% of GBR reef pixels, with 75% greater than 0.5 degrees C/week. The extent and magnitude of these SST trends are not atypical, and were greater during the same period for several other years, notably 1990 and 1993. However, compared to previous years, the early-summer combination of increasing SST trends with unprecedented levels of extensive HotSpots and DHWs are priming the GBR region for coral bleaching-level DHWs later during the 2021-2022 summer.

**Table 1.** Summary of annual heat stress conditions from 1985 to 2021. For each year, as of 14 December, the El Niño Southern Oscillation (ENSO) status (October-December) including the NINO3.4 temperature (degrees C); if there was an occurrence of a mass bleaching event on the Great Barrier Reef (GBR) during the following summer; the percentage of the 5,274 GBR 5 km satellite pixels that had a HotSpot > 1; the percentage of GBR pixels with a Degree Heating Week (DHW) > 2; and the mean background temperature for all GBR pixels. Cells within the table containing ‘--’ indicate a value of between 0.0% to 0.1% of the GBR pixels. *[Note: La Niña conditions present in December 2016 did not persist past January 2017; bleaching did not commence until February 2017.]*

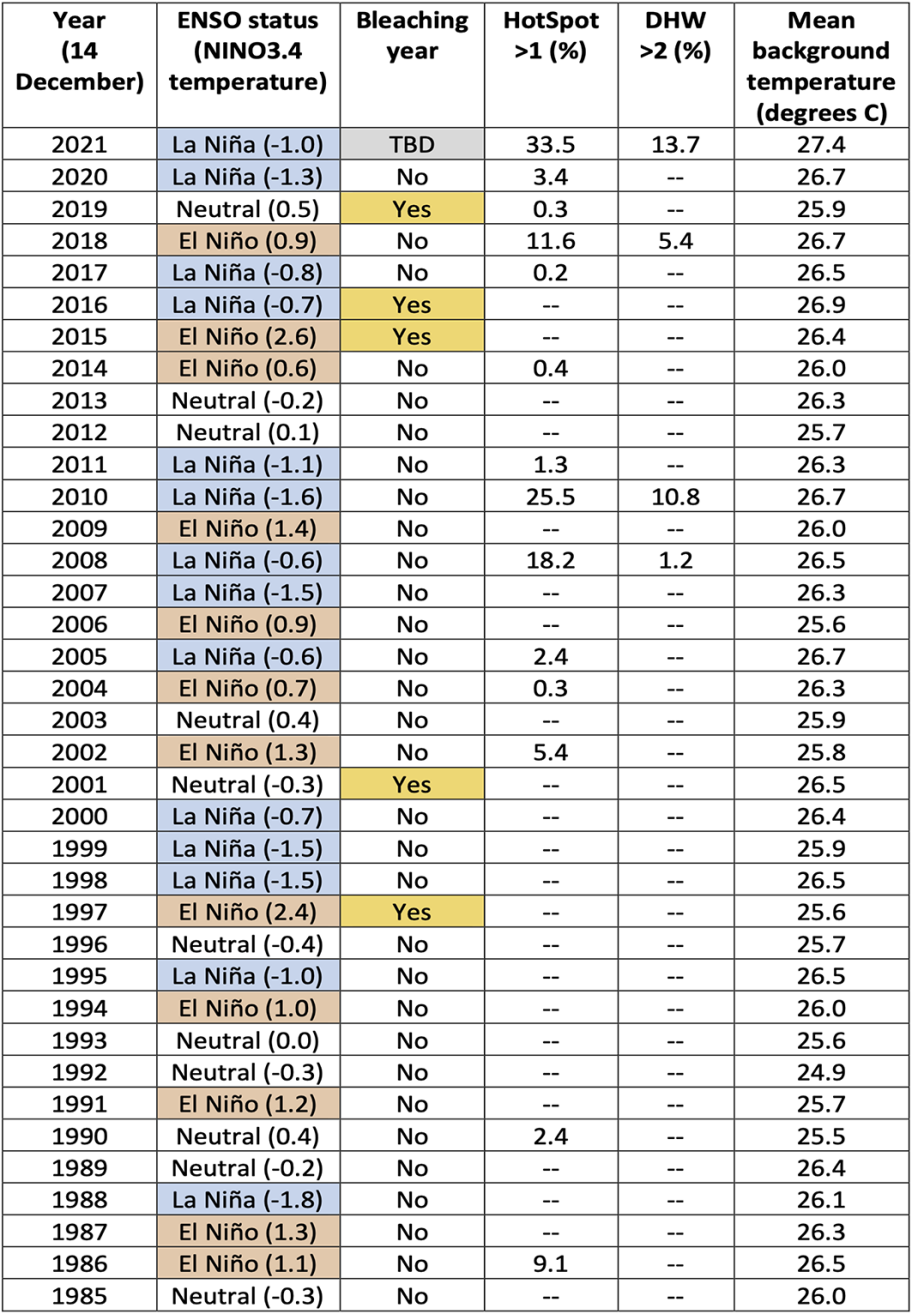

**Figure 1.**
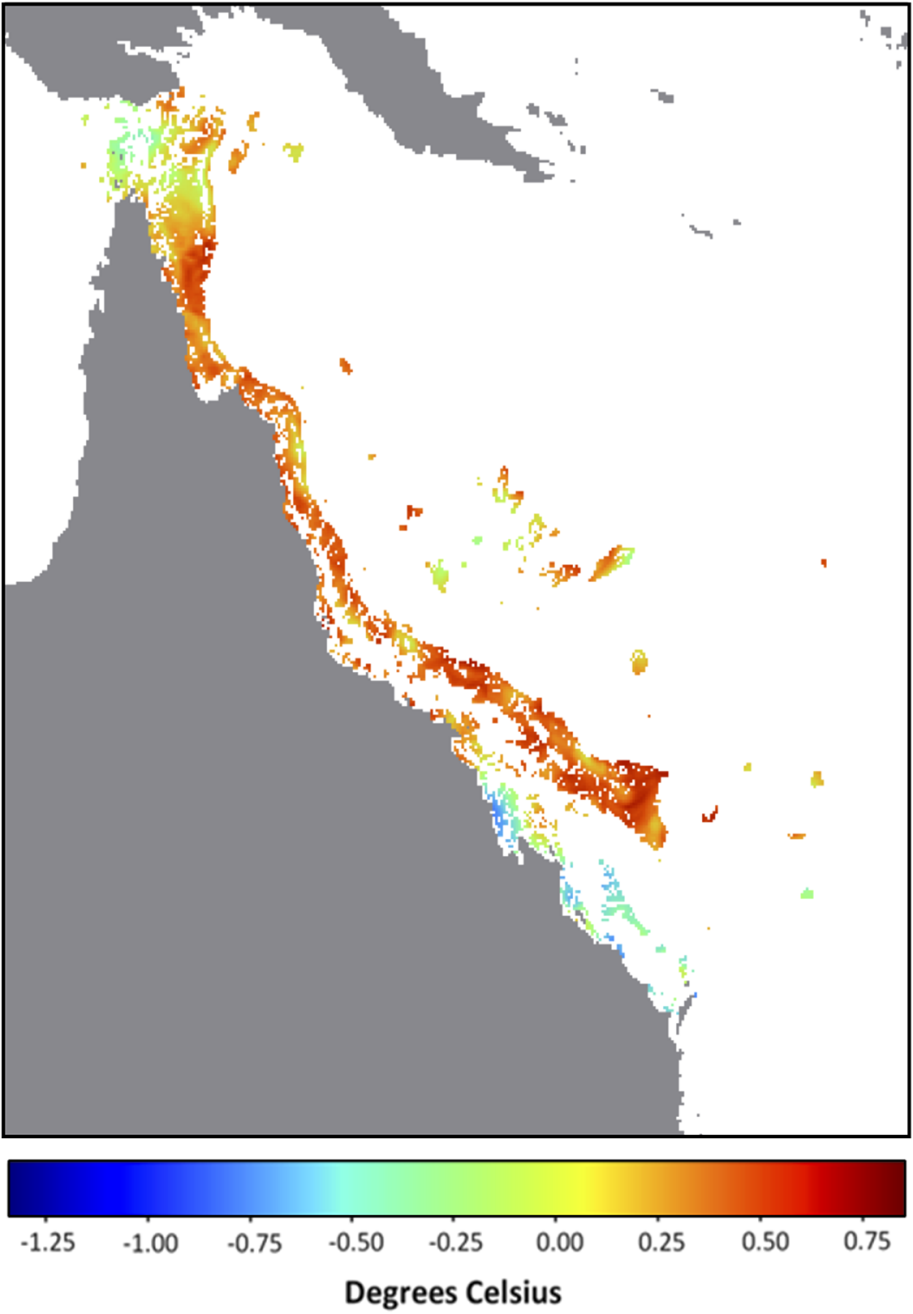
December 2021 Background temperature compared with November – December all-time maximum temperature. Map of Great Barrier Reef 5 km-resolution satellite pixels (0.05° × 0.05°) denoting the difference between the background temperature for 14 December 2021 (minimum SST observed over the previous 29 days, 16 November - 14 December) and the all-time (1985-2020) maximum temperature between 16 November and 14 December. Values indicate difference in degrees Celsius; positive values and warm colours indicate reef pixels in which the 2021 background temperature is higher than the all-time maximum.

**Figure 2.**
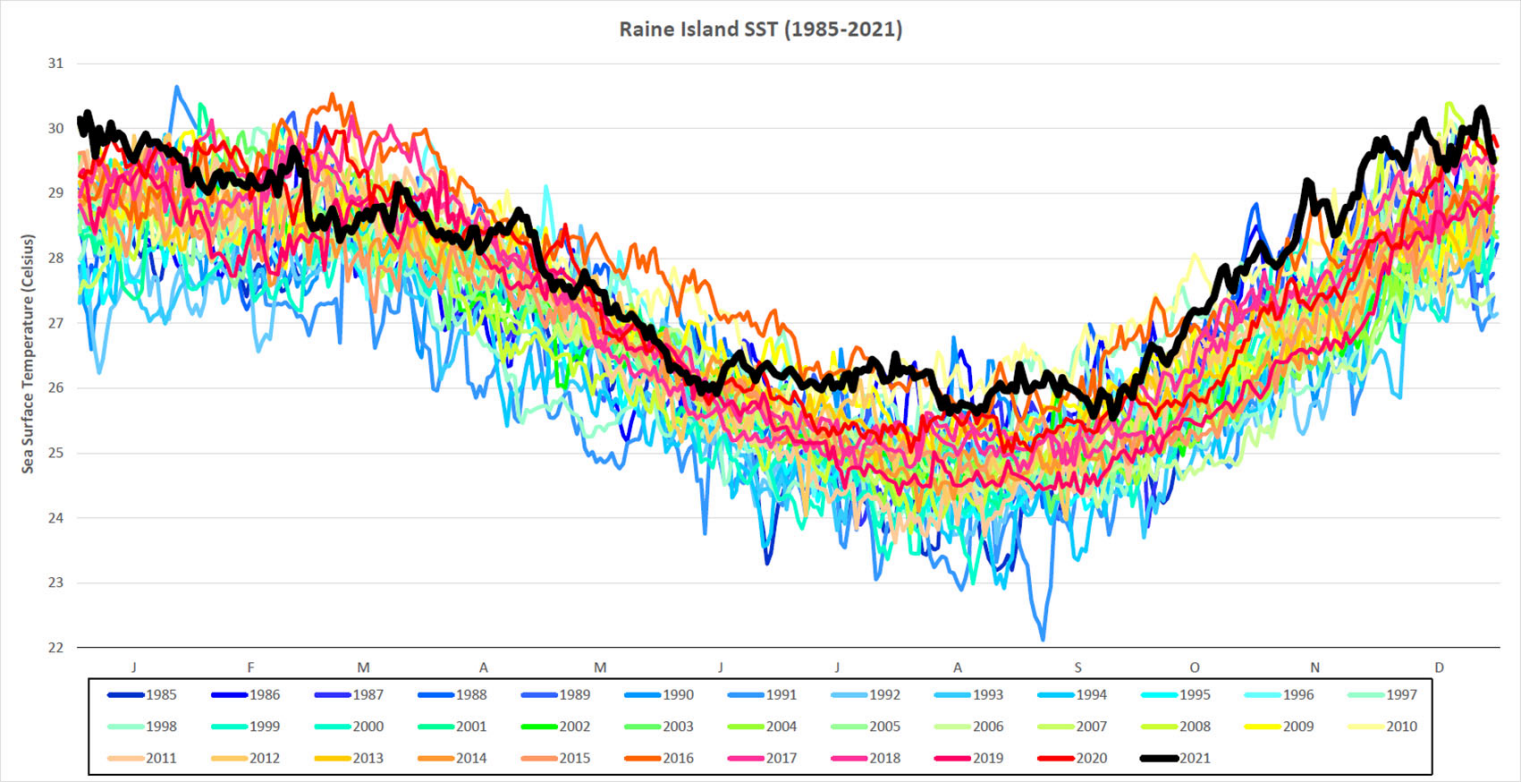
Daily SST for Raine Island from 1985-2021. Daily SST (degrees Celsius) from 1985-2021 for a single 5 km-resolution satellite pixel (0.05° × 0.05°) located in the northern Great Barrier Reef, Raine Island (11.589°S, 144.035°E). SST for 2021 is in black and in bold.

It is possible that the underlying catalyst for the developing anomalous SST conditions lies in the background temperature. In this context, we define the background temperature as the minimum temperature during the previous 29 days (period of a tidal cycle). The background temperatures on the GBR for 14 December 2021 were overwhelmingly the highest across the entire satellite SST record (i.e., compared with the minimum temperature for each pixel within the 29 days prior to and including 14 December of all years from 1985 to 2020). In fact, for 87% of GBR reef pixels, the minimum temperature at each of these pixels from 16 November to 14 December 2021 was greater than the maximum temperature at the corresponding pixel for any day between 16 November and 14 December from 1985 to 2020 ( Figure 1). This is even more noteworthy considering that during November 2021, Queensland, Australia experienced rainfall 136% above the 1961-1990 average, making it the wettest (and most likely the most intensively cloud covered with minimal surface solar heating) November since 2010 ( Australian Bureau of Meteorology). Despite this, the SST and corresponding heat stress conditions on the GBR by mid-December 2021 exceeded, in intensity and extent, that seen for any prior year, including bleaching years ( Table 1). This suggests that excessive heat energy is being caused by sources other than direct solar heating. Annual SST trends will need to be considered to understand why early-summer 2021 stands out as the warmest on record for the GBR ( Figure 2).

### Bleaching Outlook for the 2021-2022 summer season on the GBR

The NOAA CRW modelled weekly global Four-Month Coral Bleaching Heat Stress Outlook product has been generating weekly forecasts of bleaching-level heat stress conducive to mass coral bleaching since July 2011 ( Eakin *et al.*, 2012; Liu *et al.*, 2018). Using SST forecasts from the NOAA/National Weather Service/National Centers for Environmental Prediction’s Climate Forecast System Version 2 (CFSv2) ( Saha *et al.*, 2014), the CRW Outlook product predicts the likelihood of coral bleaching-level heat stress, on subseasonal-to-seasonal scales, up to four months into the future. The CFSv2 is an operational, dynamical, fully coupled ocean-land-atmosphere seasonal climate global forecast model system. CRW’s CFS-based Four-Month Coral Bleaching Outlook is detailed in Eakin *et al*. (2012) and Liu *et al*. (2018). Weekly Outlooks generate forecasts of heat stress (HotSpot and DHW) for each of the subsequent 20 weeks at a spatial resolution of 0.5° × 0.5° (approximately 50 km × 50 km). The Outlook product forecasts each 50 km ocean pixel to be in one of five stress level categories, with a corresponding potential bleaching intensity, listed in Table 2. Note that the Outlook system applies a much more complicated algorithm (described in Liu *et al.*, 2018) based on the relationship between CRW’s satellite HotSpots and DHWs as well as modelled HotSpots and DHWs. Forecasts are available in ten pre-set probabilistic levels ranging from 10% to 100% in increments of 10%. Henceforth, when referring to Outlook forecasts, we are referring to the 90% probabilistic Outlook level.

**Table 2.** Description of Stress Level categories used in Coral Reef Watch’s (CRW) satellite monitoring and modelled Four-Month Coral Bleaching Outlook products. ‘Definition for satellite monitoring’ column details the conditions necessary to meet each Stress Level category. The ‘Potential bleaching intensity’ column details the expected outcome for corals subject to each Stress Level.

Stress Level category	Definition for satellite monitoring	Potential bleaching intensity
No Stress	HotSpot ≤ 0	No bleaching
Bleaching Watch	0 < HotSpot < 1	--
Bleaching Warning	1 ≤ HotSpot and 0 < DHW < 4	Possible bleaching
Bleaching Alert Level 1	1 ≤ HotSpot and 4 ≤ DHW <8	Significant bleaching likely
Bleaching Alert Level 2	1 ≤ HotSpot and 8 ≤ DHW	Severe bleaching and significant mortality likely

On 14 December 2021, NOAA CRW’s near real-time satellite monitoring indicated that nearly the entirety of the GBR (98% of all GBR reef pixels) was at Bleaching Watch or higher, with 42% of all GBR reef pixels, including large portions of the far northern GBR, under Bleaching Warning. At a more conservative probability of 90%, Outlook forecasts for 23 January 2022, generated on 21 December 2021, predicted 20% of GBR reef pixels to be at Bleaching Alert Level 1, the majority of these north of Celebration Reef (13.283°S). By 13 February 2022, 45% of GBR reef pixels were predicted to be at Bleaching Alert Level 1 or higher. Bleaching Alert Level 2 conditions were forecast for 14% of the GBR pixels by this date. CRW’s Outlook has been forecasting heat stress for the upcoming GBR summer season earlier than ever observed previously; these forecasts are supported by the Australian Bureau of Meteorology as well as subsequent CRW satellite observations.

### GBR bleaching and ENSO state

It is noteworthy that CRW’s Outlook has forecast unprecedented heat stress for summer 2021-2022 on the GBR despite the presence of a La Niña. If the Outlook forecasts prove to be accurate, this will be the first mass coral bleaching event on the GBR during a La Niña. Two mass bleaching events on the GBR occurred during El Niño events (1998 and 2016), and three (2002, 2017, and 2020) during ‘neutral’ phases of the ENSO. The GBR bleaching event of 2017 occurred immediately following a La Niña period, but the ENSO had shifted back to a ‘neutral’ phase before bleaching commenced. Since 1985, there have been 13 La Niña, 13 ‘neutral’ and 11 El Niño states during the January/February/March period according to the Oceanic Niño Index produced by NOAA ( NOAA National Weather Service Climate Prediction Center).

The ENSO conditions for 2022 are predicted to remain in a La Niña state throughout at least January by all seven of the major international climate models. Four of these models continue the event into February, and one forecasts the La Niña to last through to April ( Australian Bureau of Meteorology; Columbia Climate School International Research Institute for Climate and Society). A La Niña is typically associated with atmospheric instability over the GBR, which is conducive to increased cloud cover, precipitation and higher winds ( Braganza, 2008; Chung and Power, 2017). The second most severe early-summer SST conditions observed on the GBR occurred in 2010 ( Table 1), as part of the 2010-2011 GBR summer season, which also coincided with a La Niña. However, these warm ocean conditions contributed to northeast Australia experiencing high cloud cover and extreme rainfall ( Evans and Boyer-Souchet, 2012; Ummenhofer *et al.*, 2015). This resulted in reduced heat stress on the GBR ( Leahy *et al.*, 2013; Zhao *et al.*, 2021), although the resultant flooding contributed to other forms of coral stress (e.g. Jones and Berkelmans, 2014). CRW’s Outlook forecasts account for the effects of La Niña. However, like other seasonal forecasts, the Outlook does not have an ability to predict the development of cyclones or very large storms. As a result, effects of these major storms cannot be included within the Outlook forecasts until they have occurred. This is an important factor to consider since cyclones have been a major mitigating and feedback factor for heat stress on the GBR in the past. While cyclones could remove some heat from the system, it is unclear whether their impact would be enough to help the GBR avoid another mass bleaching event in early 2022 due to the significant amount of early-summer heat already present on the reef and surrounding seas. As of 21 December 2021, CRW’s Outlook predicts bleaching on the GBR to commence by early January and to be widespread along the GBR by 30 January 2022.

## Conclusions

The lead-up to the 2021-2022 summer season on the GBR was uniquely warm. The extent and magnitudes of HotSpots and DHWs on GBR satellite reef pixels exceed those observed during this period in any previous year on record (1985-2020). The presence of a La Niña, and more specifically high cloud cover, could provide the GBR with some relief from heat stress ( Zhao *et al.*, 2021). Whether that would be enough to avoid bleaching-level heat stress across large sections of the GBR in January/February/March 2022 remains to be seen. For more than 85% of the GBR, the background temperatures (minimum SST over previous 29 days) for 14 December 2021 were higher than the maximum temperatures observed over the same 29-day period for any year since 1985. Therefore, it is likely that the predicted marine heatwave has been largely driven by the long-term shifts in oceanic conditions. Future studies must investigate the oceanic and climatic patterns contributing to the exceptionally warm early-summer conditions in the Coral Sea for late 2021. There is a potential for future summers to get warmer earlier and to stay warmer for longer. If this becomes a trend, it could be detrimental to the health, recovery and survival of corals on the GBR.

## Methods

SST data and heat stress metrics (HotSpots, DHWs, and seven-day SST trends) were derived from the daily global 5 km (0.05° × 0.05°) NOAA CRW Heat Stress Monitoring Product Suite version 3.1 dataset. To determine which pixels correspond with coral reefs, a global 5 km reef-pixel dataset was used to overlay the SST and heat stress metric datasets ( Heron *et al.*, 2016). All data extraction and analyses were performed in Python version 3.6.9.

The initial occurrence of DHW values greater than 3 and 4 degree C-weeks on the GBR were determined by analysing daily DHWs on GBR reef pixels for each 12-month period, starting on 1 July (Southern Hemisphere winter), within the satellite record (1985-2021). The initial occurrence for each year was defined as the dates that the target DHW values (3 and 4 degree C-weeks) or greater were observed on a GBR reef pixel. The proportion of GBR reef pixels having HotSpot values greater than 0.5 °C and 1.0 °C, as well as with DHW values greater than 2 degree C-weeks was determined for 14 December of each year in the satellite record. The seven-day SST trend of GBR reef pixels for 14 December of each year was also extracted and compared.

We defined the background temperature for a given date as the minimum SST for a pixel over the 29 days prior to and including the given date, which is the period of a full tidal cycle. The background temperature for 14 December (minimum SST from 16 November to 14 December) for each year, 1985-2021, was determined for each GBR reef pixel. The all-time maximum SST from 1985-2020 over the same 29-day period was also determined for each GBR pixel. This was then used to compare background temperatures among years as well as to determine the proportion of GBR pixels in which the background temperature on 14 December 2021 was greater than the maximum SST between 16 November and 14 December for all years from 1985-2020.

## Data availability

Sea surface temperature data and heat stress metrics used in this report are available from https://coralreefwatch.noaa.gov/product/5km_v3.1 and are archived at the NOAA National Centers for Environmental Information. Data repositories for the individual metrics used here are listed below.

*CoralTemp (Sea Surface Temperature) version 3.1:* https://www.star.nesdis.noaa.gov/pub/sod/mecb/crw/data/5km/v3.1_op/nc/v1.0/daily/sst/

*HotSpot version 3.1:* https://www.star.nesdis.noaa.gov/pub/sod/mecb/crw/data/5km/v3.1_op/nc/v1.0/daily/hs/

*Degree Heating Week version 3.1:* https://www.star.nesdis.noaa.gov/pub/sod/mecb/crw/data/5km/v3.1_op/nc/v1.0/daily/dhw/

*7-day Sea Surface Temperature Trend version 3.1:* https://www.star.nesdis.noaa.gov/pub/sod/mecb/crw/data/5km/v3.1_op/nc/v1.0/daily/sst-trend-7d/

*Four-Month Coral Bleaching Outlook version 5:* https://www.star.nesdis.noaa.gov/pub/sod/mecb/crw/data/outlook/v5/nc/v1/outlook/
