# Supplementary material for: Unprecedented early-summer heat stress and forecast of coral bleaching on the Great Barrier Reef, 2021-2022
Source: F1000Res. 2022 Nov 29;11:127. Originally published 2022 Feb 1. [Version 4] doi: 10.12688/f1000research.108724.4 (PMC9652503; doi:10.12688/f1000research.108724.4)
Supplement: Peer review history

# Peer review: Reviewer response for version 3

**DOI:** 10.5256/f1000research.134629.r152805

**Published:** 2022-11-11

**Authors:** John D Hedley

**Affiliations:** 1 Numerical Optics Ltd, Tiverton, UK

This paper is a largely descriptive discussion of trends in various satellite derived thermal metrics over the Great Barrier Reef, such as SST (sea surface temperature) and DHW (degree heating weeks, an accumulated metric of temperature excess over climatologically expected values). In my reading, the main point of the paper is that as of December 2021, under various interpretations, these metrics were unprecedentedly high on the GBR with respect to the satellite data record.

 Where there seems to be some debate is in the implied certainty of the link between these thermal metrics and coral bleaching and/or coral "stress". The paper uses terms such as "heat stress" and the product name "Coral Bleaching Heat Stress Outlook" which does imply a direct causal and predictive link between elevated temperature, coral bleaching and coral "stress". It is not in question that there is a link between elevated temperatures and coral bleaching but clearly the picture is more complicated, other factors are important and elevated temperatures may or may not lead to bleaching in certain locales and vice versa. Nevertheless the basic link is a reasonable assumption at first order. The purpose of this paper is primarily to draw attention to the elevated metrics and I don't think it is very contentious to imply this may lead to coral bleaching.

 However I would suggest the authors add a few sentences, maybe towards the end of the discussion, to make the point that these thermal metrics are not the whole story. I suggest to cite a few examples from the literature (or elsewhere) of situations in which bleaching might have been predicted from thermal metrics and didn't occur, or vice-versa. This would add more balance and would certainly be of interest to the reader.

Is the work clearly and accurately presented and does it cite the current literature?

Yes

If applicable, is the statistical analysis and its interpretation appropriate?

Yes

Are all the source data underlying the results available to ensure full reproducibility?

Yes

Is the study design appropriate and is the work technically sound?

Yes

Are the conclusions drawn adequately supported by the results?

Yes

Are sufficient details of methods and analysis provided to allow replication by others?

Yes

Reviewer Expertise:

Radiative transfer modelling, shallow water remote sensing.

I confirm that I have read this submission and believe that I have an appropriate level of expertise to confirm that it is of an acceptable scientific standard.

# Response

**Published:** 2022-11-15

**Authors:** Blake Spady

**Affiliations:** National Oceanic and Atmospheric Administration, Australia

We thank the reviewer for their comments, and greatly appreciate the time and effort that they provided to deliver the valued feedback and suggestions for our manuscript. We have included some lines at the end of the Results and Discussion section which point out that additional factors could mitigate or exacerbate the effects of heat stress, and that bleaching is not always a certainty based on heat stress metrics alone. We are hopeful that these included statements will sufficiently highlight that thermal metrics are not the whole story, but are nonetheless an important and valuable tool for monitoring our coral reefs in a changing world.

# Peer review: Reviewer response for version 2

**DOI:** 10.5256/f1000research.134147.r138337

**Published:** 2022-05-25

**Authors:** Timothy R. McClanahan

**Affiliations:** 1 Global Marine Programs, Wildlife Conservation Society, Bronx, NY, USA

This version is clearer about the heat stress forecast metrics, less frequently but still conflating heat stress with bleaching, and uses less catastrophic language. These are all good changes. There are still many problems with rigour of language and actual responses that could be improved. The language should be kept to the description of what is being measured and less about the expected response.

 For example, I think they should refer to their Four-Month Coral Bleaching Heat Stress Outlook Product, as just a Four-Month Excess Heat Outlook Metric to avoid the weak inference between heat stress and actual coral bleaching responses. For example, remember many corals do not bleach but still die under heat stress among the other bleach and die response options. One could remove bleaching from this product name and still be just as effective but more objective in communications. 

 Again, it is not that clear if these "five mass bleaching events" are based only on DHW and hotspot metrics or confirmed bleaching events or false positive predictions. If bleaching, on what scale or what metric, which species were bleached, did the event leave stress bands, etc? For example, the 2016-2017 event did not produce stress bands despite have DHW>8 (DeCarlo et al. 2019) ^1^. Other years have stress bands with DHW <4. This type of mismatch between the product or metric used here and measured responses is problematic and susceptible to selectivity in determining what are and what are not mass bleaching events. Yet, table 1 defines bleaching as DHW>2 not by observations of corals but a mostly unsupported inference. Why not just report the years with >2 DHW and avoid calling it a bleaching year. What is gained by using this type of repeatedly weak and falsified inference?

 This, like most similar papers, are overly reliant on 1 or a few highly related metrics, now considered "a product". Why were other options not evaluated, such as rate of rise, various measures of variability, light, etc? An over focus on a single metric is largely outdated by modern statistical approaches where many metrics are hypothesized, evaluated, and competed for prediction.

 Why use the economic language of product for what is a hypothesis or metric, especially one that has weak inference and been falsified many times? The product is not very good and might be returned for a refund. I jest because this is inappropriate use of language in science, in my opinion. 

 The authors are fortunate that, in this case, there was bleaching observed by plane observation. It would be useful to know how many places exhibited false positives or false negatives as per DHW threshold predictions? How might this change over a broader region outside of the GBR? Remember, the larger the scale of the analysis, the more likely there will be correspondence, so it is important to keep the scale of the DHW matched to the actual scale of bleaching. To avoid broad sweeps of confirmation. And, to define the heat response as loss of colour, stress bands, coral mortality, etc. 

 Bleaching has begun to loss its scientific meaning as it is currently used without specific responses to specific corals in specific locations. This might be fine for common conversations but less acceptable as science. This study just adds to the dilution of meaning and sense making. 

 Knowing the large oceanographic context of this study would be quite useful to understanding what caused an unusually warm year during this "triple-dip La Nina".

Is the work clearly and accurately presented and does it cite the current literature?

No

If applicable, is the statistical analysis and its interpretation appropriate?

No

Are all the source data underlying the results available to ensure full reproducibility?

Partly

Is the study design appropriate and is the work technically sound?

Partly

Are the conclusions drawn adequately supported by the results?

Partly

Are sufficient details of methods and analysis provided to allow replication by others?

Yes

Reviewer Expertise:

Coral reef ecology

I confirm that I have read this submission and believe that I have an appropriate level of expertise to confirm that it is of an acceptable scientific standard, however I have significant reservations, as outlined above.

## References

[1] : Acclimatization of massive reef-building corals to consecutive heatwaves. *Proceedings of the Royal Society B: Biological Sciences* .2019;286(1898) : 10.1098/rspb.2019.0235 10.1098/rspb.2019.0235 PMC645833330836872

# Response

**Published:** 2022-05-29

**Authors:** Blake Spady

**Affiliations:** National Oceanic and Atmospheric Administration, Australia

Thank you for your additional comments and feedback. Please find our response to each comment below

***Comment 1:***

*This version is clearer about the heat stress forecast metrics, less frequently but still conflating heat stress with bleaching, and uses less catastrophic language. These are all good changes. There are still many problems with rigour of language and actual responses that could be improved. The language should be kept to the description of what is being measured and less about the expected response.*

**Response 1:**

 We can appreciate your general concerns regarding the language used in relation to heat stress and coral bleaching. However, we feel obliged to point out that the changes you are describing in the above comment have not occurred within this manuscript so it is difficult to understand what specific issues you are referencing here. A change made from version 1 to the current version of this manuscript was a rephrasing of the definition of mass coral bleaching related to excess sea surface temperature (SST), as suggested by reviewer #2 (Introduction, 1 ^st^ paragraph, 3 ^rd^ sentence). We therefore find it impossible to interpret exactly which language problems you have with the current or former manuscript due to a lack of details. We have, however, included in this revised version a statement at the end of the Results and Discussion section highlighting the limitations of the CRW Outlook product. This statement reads:

 “It is important to note that the predictions from CRW’s Outlook product described in this study are being used to provide a general warning for large sections of the GBR, and do not aim to predict bleaching for individual pixels or reefs.”

***Comment 2:***

*For example, I think they should refer to their Four-Month Coral Bleaching Heat Stress Outlook Product, as just a Four-Month Excess Heat Outlook Metric to avoid the weak inference between heat stress and actual coral bleaching responses. For example, remember many corals do not bleach but still die under heat stress among the other bleach and die response options. One could remove bleaching from this product name and still be just as effective but more objective in communications.*

**Response 2:**

 The Coral Reef Watch (CRW) Four-Month Coral Bleaching Heat Stress Outlook Product is an official National Oceanic and Atmospheric Administration (NOAA) product; we merely referred to it by using its official name, which was not decided on when crafting this manuscript.

***Comment 3:***

*Again, it is not that clear if these "five mass bleaching events" are based only on DHW and hotspot metrics or confirmed bleaching events or false positive predictions. If bleaching, on what scale or what metric, which species were bleached, did the event leave stress bands, etc? For example, the 2016-2017 event did not produce stress bands despite have DHW>8 (DeCarlo et al. 2019)* *^1^* *. Other years have stress bands with DHW <4. This type of mismatch between the product or metric used here and measured responses is problematic and susceptible to selectivity in determining what are and what are not mass bleaching events. Yet, table 1 defines bleaching as DHW>2 not by observations of corals but a mostly unsupported inference. Why not just report the years with >2 DHW and avoid calling it a bleaching year. What is gained by using this type of repeatedly weak and falsified inference?*

**Response 3:**

 The five mass bleaching events described in the Introduction section of this paper are observed and well documented events on the Great Barrier Reef (GBR). Our three statements specifically referring to these events do not speak to the Degree Heating Week (DHW) values, nor other heat stress metrics, associated with those events. We simply reference their occurrence and point out that three of the five events occurred between 2016 and 2020. In relation to these heat stress events, we do cite Hughes et al. (2021), which describes the mass bleaching events and analyses many of the heat stress metrics and other factors you are hoping to see here. These analyses are beyond the scope and aim of this study.

 Further, regarding your statement about Table 1 in our manuscript, nowhere in Table 1, nor within this paper, do we define bleaching as DHW>2. We can only assume you are referencing the fifth column of Table 1 (with the header “DHW>2 (%)”). This is very clearly described as quantifying the percentage of the 5,274 GBR reef pixels (0.05 x 0.05 degrees) which had a DHW value greater than 2 as of 14 ^th^ December in each of the listed years. Values in the “DHW>2 (%)” column are not used to define any bleaching event, rather they are for comparing the differences between the years. In fact, the third column of Table 1 (with the header “Bleaching year”) specifies which years had observed mass bleaching events on the GBR following the December period. None of the years preceding mass bleaching events (i.e. those labelled “yes” in the “Bleaching year” column) correspond with a presented value for percentage of pixels with DHW >2 in the fifth column. Therefore, we are unclear how you came to that conclusion. Table 1 describes plainly the unprecedented early-summer heat stress conditions on the GBR for late 2021, comparing to previous years, and makes no predictions of mass or other coral bleaching.    

***Comment 4:***

*This, like most similar papers, are overly reliant on 1 or a few highly related metrics, now considered "a product". Why were other options not evaluated, such as rate of rise, various measures of variability, light, etc? An over focus on a single metric is largely outdated by modern statistical approaches where many metrics are hypothesized, evaluated, and competed for prediction.*

**Response 4:**

 We do not consider this study to be “overly reliant” on a single metric. Here, we simply state the facts of heat stress metrics as observed in the early-summer on the GBR. As mentioned in our previous response, we understand that many factors contribute to coral bleaching in general, but that accumulated heat stress has a much stronger correlation to instances of mass coral bleaching. As such, we observed unprecedented heat stress accumulation in late 2021, and forecasts of heat stress accumulation exceeding that which has been known to correlate with mass coral bleaching on the GBR in the recent past. We took this opportunity to describe these conditions as they were unfolding, and to warn scientists, managers, and other stakeholders who work on the GBR of the potential for another mass bleaching event.

 Furthermore, CRW’s satellite sea surface temperature-based monitoring metrics are the only ones available and produced operationally for coral reefs globally, including the GBR. Our study took advantage of the metrics that are commonly used by and available to the global coral reef communities in near real-time and on an operational basis for the purpose of continuous monitoring of the thermal conditions during the entire 2021-2022 GBR summer season. This article does not aim to develop new products for monitoring and predicting bleaching. We intended to use only the data that were available to both us and the rest of coral reef community at the time of analysis and beyond. We are interested in incorporating other parameters and metrics, including those you mentioned; however, none of those are available operationally and in near real-time.

***Comment 5:***

*Why use the economic language of product for what is a hypothesis or metric, especially one that has weak inference and been falsified many times? The product is not very good and might be returned for a refund. I jest because this is inappropriate use of language in science, in my opinion.*

**Response 5:**

 The established terminology and naming of metrics is not a matter appropriately explored within this study. The term “product” is used by NOAA and many other agencies in the U.S. and internationally to describe the various operational satellite-based outputs produced for public use, hence our use of the term. This term has been used for decades before the writing of this paper commenced, and we do not find it within the scope of this study to attempt to change that.

***Comment 6:***

*The authors are fortunate that, in this case, there was bleaching observed by plane observation. It would be useful to know how many places exhibited false positives or false negatives as per DHW threshold predictions? How might this change over a broader region outside of the GBR? Remember, the larger the scale of the analysis, the more likely there will be correspondence, so it is important to keep the scale of the DHW matched to the actual scale of bleaching. To avoid broad sweeps of confirmation. And, to define the heat response as loss of colour, stress bands, coral mortality, etc.*

**Response 6:**

 You may categorise the 2021-2022 mass bleaching event on the GBR as a fortunate outcome for us, however, we take no pleasure in the fact that 91% of the 719 reefs surveyed by the Great Barrier Reef Marine Park Authority were found to have some level of coral bleaching, with large areas experiencing over 60% coral cover bleached ( https://www.gbrmpa.gov.au/the-reef/reef-health). Also, we were confident that the level of accumulated heat stress forecasted was capable of resulting in a mass bleaching event on the GBR, and that has been shown to be correct. We do not view this as us having good luck, but rather as supporting evidence that the extent and degree of accumulated heat stress detailed is sufficient to cause mass coral bleaching on the GBR.

 We do, however, agree that it would be useful to know how many reefs exhibited false positives and false negatives during this event. This is something that we plan to look at in the future, when the bleaching observation data from this event is made available, and we encourage others to explore this as well. This question is beyond the scope of the current study and is also not possible until the bleaching observation data from this event are collated and released. Please note that the intent of this paper is to provide an overall warning of mass coral bleaching for the GBR, and does not aim to provide a warning for any individual reefs, or pixels, within the GBR.

    

***Comment 7:***

*Bleaching has begun to loss its scientific meaning as it is currently used without specific responses to specific corals in specific locations. This might be fine for common conversations but less acceptable as science. This study just adds to the dilution of meaning and sense making.*

*Knowing the large oceanographic context of this study would be quite useful to understanding what caused an unusually warm year during this "triple-dip La Nina".*

**Response 7:**

 Coral bleaching is a very commonly used term worldwide and we will not take issue with that term in the context of this paper. Regardless, in the Introduction, we have clearly defined “coral bleaching” as a stress response which disrupts the symbiosis between corals and their endosymbiotic algae (zooxanthellae), causing the corals to expel zooxanthellae. We have also defined “mass coral bleaching” as bleaching at a scale of an entire reef system or geographic realm. We have clarified that, for the purposes of this study, we are only focusing on mass coral bleaching and not bleaching more generally. While we agree that, in general, the process of coral bleaching could be better understood with more nuanced terminology and definitions, we have made it clear what we consider mass coral bleaching to be in this context.

 Again, to reiterate from our responses to your initial review of this manuscript, we agree that an understanding of the larger oceanic context of this unprecedented early-summer on the GBR would be a worthy academic pursuit. However, the aim of this study is descriptive in nature, and sought to specifically detail the observed and forecasted conditions present on the GBR only. The academic pursuit you suggest is beyond the scope of this study, but we do encourage others to explore this. We must also point out that the Conclusion section all submitted versions of this manuscript suggest exactly that by stating:

 “Therefore, it is likely that the predicted marine heatwave has been largely driven by the long-term shifts in oceanic conditions. Future studies must investigate the oceanic and climatic patterns contributing to the exceptionally warm early-summer conditions in the Coral Sea for late 2021.”       

**References:**

 Hughes TP, Kerry JT, Connolly SR, et al.: Emergent properties in the responses of tropical corals to recurrent climate extremes. Curr. Biol. 2021; 31: 5393-5399.

# Response

**Published:** 2022-06-13

**Authors:** Tim McClanahan

**Affiliations:** Wildlife Conservation Society, Kenya

The language has been a problem for me as there seems to be too many assumptions about what DHW exposure does to corals. The authors are sticking to their position of bleaching and products based on history but my point is that the history of science has shown these products to not be very effective. These studies go back to 2008 and before. Writing and reporting the rates of rise and DHW is fine but the connection to bleaching is not really warranted given the many studies that show a poor connection. Particularly when projected into the future. I list some papers below but there are many others that are quite critical of the metric and the threshold for bleaching and mortality. 

 Apologies that I had not understood table 1 due to long sentences and small fonts. it took me a number of readings and reading the reviewers comments until I understood it. I think it could be better described and it is interesting how many bleaching years did not have heat conditions that would be used to predict bleaching. This failure of prediction is not mentioned in the paper but an important part of the science and objectivity that I believe is weak in the original and revisions. 

 My problem was actually with table 2 where they present the expected bleaching responses for different DHW. This table should be removed as the evidence for these relationships are not strong when they are tested in the literature. The paper does not require this table, it is in many papers and seldom supported. So, why continue to use it given all of the studies that find it is not evident. It is also becoming a problems as it is not being used to predict the future state of reefs. This is not deserved from the critical findings. 

 I think the authors should hear from a few more reviewers to know if my position is widely shared. That is if the language is assumptive and not supported by the field of thermal stress on corals or not. I believe the authors can write this paper in a less assumptive way if they make an effort. 

 Boylan, P., and J. Kleypas. 2008. New insights into the exposure and sensitivity of coral reefs to ocean warming. Pages 854-858 *in* Proceedings of the 11th International Coral Reef Symposium, Ft. Lauderdale, Florida.

 van Hooidonk, R., and M. Huber. 2009. Quantifying the quality of coral bleaching predictions. Coral Reefs **28**:579-587.

 Donner, S. D. 2011. An evaluation of the effect of recent temperature variability on the prediction of coral bleaching events. Ecological Applications **21**:1718-1730.

 Logan, C. A., J. P. Dunne, C. M. Eakin, and S. D. Donner. 2012. A framework for comparing coral bleaching thresholds. Page 10A13 *in* Proceedings of the 12th International Coral Reef Symposium, Cairns, Australia.

 DeCarlo, T. M. 2020. Treating coral bleaching as weather: A framework to validate and optimize prediction skill. PeerJ **8**:e9449.

# Response

**Published:** 2022-06-28

**Authors:** Blake Spady

**Affiliations:** National Oceanic and Atmospheric Administration, Australia

Thank you for your time and effort in providing us feedback during this review. We are sorry that we could not reach a mutual understanding here. Nevertheless, we have provided responses to each of your comments below.

***Comment 1:***

*The language has been a problem for me as there seems to be too many assumptions about what DHW exposure does to corals. The authors are sticking to their position of bleaching and products based on history but my point is that the history of science has shown these products to not be very effective. These studies go back to 2008 and before. Writing and reporting the rates of rise and DHW is fine but the connection to bleaching is not really warranted given the many studies that show a poor connection. Particularly when projected into the future. I list some papers below but there are many others that are quite critical of the metric and the threshold for bleaching and mortality.*

**Response 1:**

 As stated in our previous reply, we are unclear what language within our manuscript you are still taking issue with, as you have not provided specific examples for us to consider. Predictions of bleaching in the manuscript were put forward as a “potential”; we made no claims otherwise. We are not opposed to altering the manuscript text, if necessary, but we would need more direction as to where you feel issues remain. We have addressed all questions and concerns posed in your reviews, and by reviewer #2, previously versions 2 and 3 of the manuscript. The forecast of a potential for mass coral bleaching on the Great Barrier Reef (GBR) was derived strictly from NOAA’s official Four-Month Coral Bleaching Heat Stress Outlook product (henceforth referred to as the Outlook product). The descriptions of early-summer rates of rise of sea surface temperature (SST) or rates of rise of Degree Heating Weeks (DHWs) observed in November and December 2021 from the satellite data is an independent description and is not described as being connected to the Outlook forecast. The description of the unprecedented early-summer SST conditions monitored by satellite, in near real-time, on the GBR should not be conflated with the forecast of heat stress accumulation generated by the Outlook product. Further, we make no predictions of coral mortality in this study, as you seem to imply here.

 We have reviewed your list of references and are aware of them. Another suitable addition to this list could be Lachs et al. (2021), which two of this manuscript’s authors also co-authored. Lachs et al. (2021) demonstrates that certain changes to the DHW algorithm can improve global predictive skill, including dropping the Coral Bleaching HotSpot threshold to less than or equal to that of the maximum of monthly mean, and reducing the accumulation window from 12 weeks to between 4 and 8 weeks. We are all in agreement that the DHW algorithm could be improved, and could even be incorporated into a multivariate product. However, as stated previously during this review process, there are currently no other operational near real-time satellite products available for monitoring immediate heat stress that incorporate the improvements suggested here or elsewhere. Further, there are currently no other operational forecasting products available to quantify potential near-future coral stress that incorporate the improvements suggested here or elsewhere. Despite the existence of a handful of critical papers, users continue to apply and review these products and data within their own published work in peer-reviewed scientific literature. A sample of these peer-reviewed papers is available here: https://coralreefwatch.noaa.gov/satellite/publications/related_pubs_using_crw_data-products.pdf.  

 There are many ways to estimate heat stress impacts to marine organisms, including corals (Podesta and Glynn 1997; Winter et al. 1998; Manzello et al. 2007a). The DHW metric that we utilised in this paper is but one metric that estimates that accumulation of thermal anomalies above a locally calculated climatology. No matter what heat stress metric is utilised, the early-summer 2021 conditions on the GBR were unprecedentedly warm compared to previous years.

 Despite a handful of papers that have pointed out shortcomings of the DHW metric (e.g. McClanahan et al. 2007; DeCarlo 2020; McClanahan 2022), there have been many more peer-reviewed papers that have satisfactorily used DHWs as a means to estimate heat stress for corals (e.g. Manzello et al. 2007b; Couch et al. 2017; Hughes et al. 2017; Kayanne 2017; Gintert et al. 2018; Smith et al. 2019; Sully et al. 2019; Duarte et al. 2020; Spillman and Smith 2021). DHWs are used to estimate heat stress in the field, but are also the basis for the climate models that predict annual severe bleaching of coral reefs in the next two decades (Donner et al. 2009; van Hooidonk et al. 2014; van Woesik et al. 2022). In other words, DHWs are the current accepted standard for estimating heat stress on coral reefs.

 Although DHWs are the standard, we acknowledge that this metric may have issues, and, as a result, the NOAA Coral Reef Watch team have already been working on a robust scientific evaluation of the predictive capabilities of DHWs using global in situ observations of coral bleaching. This large research effort warrants its own stand-alone paper and is outside the scope of this current manuscript. We reiterate that the current manuscript is solely descriptive in nature and simply describes how SSTs were anomalously warm on the GBR prior to the bleaching season. Our additional aim was to provide a timely warning to managers, researchers and other stakeholders of the potential for mass bleaching level heat stress accumulation by the end of the summer season, which did in fact manifest into a mass bleaching event as this paper predicted. How the resultant DHWs from this event compare to the actual bleaching observations will be the focus of a future publication.   

***Comment 2:***

*Apologies that I had not understood table 1 due to long sentences and small fonts. it took me a number of readings and reading the reviewers comments until I understood it. I think it could be better described and it is interesting how many bleaching years did not have heat conditions that would be used to predict bleaching. This failure of prediction is not mentioned in the paper but an important part of the science and objectivity that I believe is weak in the original and revisions.*

**Response 2:**

 Regarding the misinterpretation of **Table 1,** the legend provides a straight-forward description; however, we welcome textual edits if they are thought necessary to improve the description. There are six variables being described in this table, the description for each being separated by semicolons. The small font, however, is beyond our control as this is managed by the typesetters within the *F1000Research *journal. You are correct that almost all of the bleaching years on the GBR had less than 0.1% of reef pixels being greater than a HotSpot value of 1 and a DHW value of 2, as of 14 ^th^ December of the corresponding summer. We agree that this is interesting, which is why we included the “Bleaching year” category within the **Table 1**. However, despite your renewed interpretation of this table, it still appears that you are suggesting these values are being used to predict bleaching, and this is not true. The values in this table are simply a summary of HotSpot and DHW values on 14 ^th^ December of all the years in our data record for the purpose of demonstrating that, in the summer of those major bleaching years, HotSpot and DHW were never has extensive as in December 2021. There is no prediction involved here as is clearly articulated in the table legend, which we have repeatedly examined based on your comments. This year-to-year comparison of the SST conditions in mid-December warrants concern on what would lie ahead for the GBR during the peak of summer 2021-2022, however, this was not what was used to make the prediction of the potential for mass bleaching level heat stress within this manuscript. This table highlights that there was unprecedented early-summer heat stress on the GBR in late 2021, and this magnitude of early-summer stress had never been reached prior to a past bleaching event. This alert of early-summer heat stress is the purpose of this paper.  

 While there is no apparent connection between early-summer DHWs and mass bleaching events on the GBR ( **Table 1**), this does not invalidate the role of DHWs in assessing the accumulated heat stress that can lead to mass coral bleaching.

***Comment 3:***

*My problem was actually with table 2 where they present the expected bleaching responses for different DHW. This table should be removed as the evidence for these relationships are not strong when they are tested in the literature. The paper does not require this table, it is in many papers and seldom supported. So, why continue to use it given all of the studies that find it is not evident. It is also becoming a problems as it is not being used to predict the future state of reefs. This is not deserved from the critical findings.*

**Response 3:**

 We are sorry you did not raise concerns with **Table 2** in your previous comments, before withdrawing from the review process. To clear up any confusion, **Table 2 **describes the heat stress level categories used in NOAA Coral Reef Watch’s operational satellite monitoring and modelled Four-Month Coral Bleaching Heat Stress Outlook product. These categories were not defined or decided upon during this study. The Outlook product categorises ocean pixels based on heat stress metrics, and provides a description titled “Potential bleaching intensity”, to relate these defined heat stress levels with the possibility of mass coral bleaching. In order to describe the forecasts generated by the Outlook product, it is necessary to understand the output metrics and the reasoning behind their categorisations. We do not suggest that these categories guarantee the outcome listed in the “Potential bleaching intensity” column under any of these scenarios. These categories are merely a description of the operational Outlook product; these are for guidance only and are not deterministic.     

***Comment 4:***

*I think the authors should hear from a few more reviewers to know if my position is widely shared. That is if the language is assumptive and not supported by the field of thermal stress on corals or not. I believe the authors can write this paper in a less assumptive way if they make an effort.*

**Response 4:**

 We genuinely thank you again for your time and effort in providing feedback for this study. We welcome your suggestion for additional reviewers and look forward to their feedback as well.

**References:**

 Couch CS, Burns JHR, Liu G, Steward K, Gutlay TN, et al. (2017) Mass coral bleaching due to unprecedented marine heatwave in Papahānaumokuākea Marine National Monument (Northwestern Hawaiian Islands). *PloS one* **12** (9), e0185121. https://doi.org/10.1371/journal.pone.0185121****

 DeCarlo TM (2020) Treating coral bleaching as weather: a framework to validate and optimize prediction skill. *PeerJ* **8**, e9449. https://doi.org/10.7717/peerj.9449

 Donner SD, Heron SF, Skirving WJ (2009) Future scenarios: a review of modelling efforts to predict the future of coral reefs in an era of climate change. *Coral Bleaching* pp. 159-173. https://link.springer.com/chapter/10.1007/978-3-540-69775-6_10

 Duarte GAS, Villela HDM, Deocleciano M, Silva D, Barno A, et al. Heat waves are a major threat to turbid coral reefs in Brazil. *Frontiers in Marine Science* **7**, 179. https://doi.org/10.3389/fmars.2020.00179

 Hughes TP, Kerry JT, Álvarez-Noriega M, Álvarez-Romero JG, Anderson KD, et al. (2017) Global warming and recurrent mass bleaching of corals. *Nature* **543**, 373-377. https://doi.org/10.1038/nature21707 

 Gintert B, Manzello DP, Enochs IC, Kolodziej G, Carlton R, Gleason A, Gracias N (2018) Marked Annual Coral Bleaching Resilience of an Inshore Patch Reef in the Florida Keys: A nugget of hope, aberrance, or last man standing? *Coral Reefs* **37**, 533-547. https://doi.org/10.1007/s00338-018-1678-x

 Kayanne H (2017) Validation of degree heating weeks as a coral bleaching index in the northwestern Pacific. *Coral Reefs* **36**, 63-70. https://doi.org/10.1007/s00338-016-1524-y

 Lachs L, Bythell JC, East HK, Edwards AJ, Mumby PJ, Skirving WJ, Spady BL, Guest JR (2021) Fine-tuning heat stress algorithms to optimise global predictions of mass coral bleaching. *Remote Sensing* 13, 2677. https://doi.org/10.3390/rs13142677   

 Manzello DP, Berkelmans RW, Hendee JC (2007a) Coral bleaching indices and thresholds for the Florida Reef Tract, Bahamas, and St. Croix, U.S. Virgin Islands. *Marine Pollution Bulletin* **54**, 1923-1931. http://dx.doi.org/10.1016/j.marpolbul.2007.08.009

 Manzello DP, Brandt M, Smith TB, Lirman D, Hendee JC, Nemeth R (2007b) Hurricanes benefit bleached corals. *Proceedings of the National Academy of Sciences, U.S.A.* **104** (29), 12035-12039. http://dx.doi.org/10.1073/pnas.0701194104

 McClanahan TR, Ateweberhan M, Ruiz Sebastian C, Graham NAJ, Wilson SK, Bruggemann JH, Guillaume MMM (2007) Predictability of coral bleaching from synoptic satellite and in situ

 temperature observations. *Coral Reefs* **26**, 695–701. https://doi.org/10.1007/s00338-006-0193-7

 McClanahan TR (2022) Coral responses to climate change exposure. *Environmental Research Letters* **17**, 073001. https://doi.org/10.1088/1748-9326/ac7478

 Podesta, G.P., Glynn, P.W., 1997. Sea surface temperature variability in Panama and Galapagos: extreme temperatures causing coral bleaching. *Journal of Geophysical Research* **102**, 15749–15759. https://doi.org/10.1029/96JC03557

 Smith KM, Payton TG, Sims RJ, Stroud CS, Jeanes RC, et al. (2019) Impacts of consecutive bleaching events and local algal abundance on transplanted coral colonies in the Florida Keys. *Coral Reefs* **38**, 851-861. https://doi.org/10.1007/s00338-019-01823-7

 Spillman CM and Smith GA (2021) A new operational seasonal thermal stress prediction tool for coral reefs around Australia. *Frontiers in Marine Science* **8**, 687833. https://doi.org/10.3389/fmars.2021.687833  

 Sully S, Burkepile DE, Donovan MK, Hodgson G, and van Woesik (2019) A global analysis of coral bleaching over the past two decades. *Nature Communications* **10**, 1264. https://doi.org/10.1038/s41467-019-09238-2

 van Hooidonk R, Maynard J, Manzello DP, Planes S (2014) Opposite latitudinal gradients in projected ocean acidification and bleaching impacts on coral reefs. *Global Change Biology* **20 **(1), 103-112. https://doi.org/10.1111/gcb.12394

 van Woesik R, Shlesinger T, Grottoli AG, Toonen RJ, Vega Thurber R, et al. (2022) Coral-bleaching responses to climate change across biological scales. *Global Change Biology* **28 **(14), 4229-4250. https://doi.org/10.1111/gcb.16192  

 Winter, A., Appeldorn, R.S., Bruckner, A., Williams Jr., E.H., Goenaga, C., 1998. Sea surface temperatures and coral reef bleaching off La Parguera, Puerto Rico (northeastern Caribbean Sea). *Coral Reefs* **17**, 377–382. https://doi.org/10.1007/s003380050143

# Peer review: Reviewer response for version 2

**DOI:** 10.5256/f1000research.134147.r138336

**Published:** 2022-05-25

**Authors:** Pedro C. González-Espinosa

**Affiliations:** 1 Institute for the Oceans and Fisheries, University of British Columbia, Vancouver, BC, Canada

I have no more comments, the point and aim of the paper/report are clear.

 As mentioned in my last comment of the first review and rephrasing it, this study focused on describing the thermal exposure using the unique operational product available to assess thermal stress, as such, it successfully reports the unprecedented conditions in the historical record

Is the work clearly and accurately presented and does it cite the current literature?

Partly

If applicable, is the statistical analysis and its interpretation appropriate?

Partly

Are all the source data underlying the results available to ensure full reproducibility?

Yes

Is the study design appropriate and is the work technically sound?

Partly

Are the conclusions drawn adequately supported by the results?

Partly

Are sufficient details of methods and analysis provided to allow replication by others?

Yes

Reviewer Expertise:

Coral reef ecology, multiple drivers.

I confirm that I have read this submission and believe that I have an appropriate level of expertise to confirm that it is of an acceptable scientific standard.

# Peer review: Reviewer response for version 1

**DOI:** 10.5256/f1000research.120142.r135987

**Published:** 2022-05-12

**Authors:** Pedro C. González-Espinosa

**Affiliations:** 1 Institute for the Oceans and Fisheries, University of British Columbia, Vancouver, BC, Canada

The authors characterized the thermal conditions of the GBR using CRW´s near real-time satellite-based heat stress products. They described an unprecedented event that hit the historical records in the onset of bleaching conditions and its magnitude.

 Taking as a reference the Stress Level categories, used in Coral Reef Watch (CRW), they forecast a mass coral bleaching in the next season. While the description is detailed, the study is limited by focusing only on thermal exposure; NOAA’s DHW tends to overpredict events of bleaching.

 Considering other factors is becoming necessary because at a regional and local scale there are several factors that could change the predictions and avoid overpredictions (error type I). For example, turbidity can exacerbate (if linked to water quality, e.g., Wooldridge 2020 ^1^) or mitigate the effects (if linked to the reduction of light stress, e.g., Sully and Van Woesik 2020 ^2^, Gonzalez-Espinosa and Donner 2021 ^3^). However, even though there is evidence that the susceptibility of coral communities to mass bleaching from thermal stress is constrained to the local biological and environmental contexts, and multivariate prediction methods that incorporate other environmental variables (e.g., solar radiation, or light attenuation) and other temperature metrics (e.g. heating rate, high-frequency variability, bimodality) have suggested an improvement in predicting the spatial variability in bleaching intensity for individual events (Maina et al., 2008 ^4^; McClanahan et al., 2019 ^5^; Safaie et al., 2018 ^6^; Sully & van Woesik, 2020 ^2^), the algorithms used to predict bleaching (as an operational tool) remain focused only on temperature.  

 Finally, I would be cautious with the assumption that mass bleaching “has only been linked to the stress of excess sea surface temperatures” because even though the elevated temperature is often reported as the primary cause of observed mass coral bleaching, it is the interaction between temperature and other environmental variables that modulate bleaching responses. A quick exploratory analysis of other environmental variables (e.g., description or time series of turbidity, cloudiness, etc) could be included in the discussion.

 On the other hand, since this study is focused on describing the thermal exposure using a single metric approach then it successfully reports the unprecedented conditions in the historical record.

Is the work clearly and accurately presented and does it cite the current literature?

Partly

If applicable, is the statistical analysis and its interpretation appropriate?

Partly

Are all the source data underlying the results available to ensure full reproducibility?

Yes

Is the study design appropriate and is the work technically sound?

Partly

Are the conclusions drawn adequately supported by the results?

Partly

Are sufficient details of methods and analysis provided to allow replication by others?

Yes

Reviewer Expertise:

Coral reef ecology, multiple drivers.

I confirm that I have read this submission and believe that I have an appropriate level of expertise to confirm that it is of an acceptable scientific standard, however I have significant reservations, as outlined above.

## References

[1] : Excess seawater nutrients, enlarged algal symbiont densities and bleaching sensitive reef locations: 1. Identifying thresholds of concern for the Great Barrier Reef, Australia. *Mar Pollut Bull* .2020;152: 10.1016/j.marpolbul.2016.04.054 107667 10.1016/j.marpolbul.2016.04.054 27230986

[2] : Turbid reefs moderate coral bleaching under climate-related temperature stress. *Glob Chang Biol* .26(3) : 10.1111/gcb.14948 1367-1373 10.1111/gcb.14948 31912964PMC7079097

[3] : Cloudiness reduces the bleaching response of coral reefs exposed to heat stress. *Glob Chang Biol* .27(15) : 10.1111/gcb.15676 3474-3486 10.1111/gcb.15676 33964101

[4] : Modelling susceptibility of coral reefs to environmental stress using remote sensing data and GIS models. *Ecological Modelling* .2008;212(3-4) : 10.1016/j.ecolmodel.2007.10.033 180-199 10.1016/j.ecolmodel.2007.10.033

[5] : Temperature patterns and mechanisms influencing coral bleaching during the 2016 El Niño. *Nature Climate Change* .2019;9(11) : 10.1038/s41558-019-0576-8 845-851 10.1038/s41558-019-0576-8

[6] : High frequency temperature variability reduces the risk of coral bleaching. *Nature Communications* .2018;9(1) : 10.1038/s41467-018-04074-2 10.1038/s41467-018-04074-2 PMC592011429700296

# Response

**Published:** 2022-05-17

**Authors:** Blake Spady

**Affiliations:** National Oceanic and Atmospheric Administration, Australia

We greatly appreciate the time and effort that the reviewer has provided to deliver the valued feedback and suggestions for our manuscript. Please find our response to each comment below.

***Comment 1:***

*The authors characterized the thermal conditions of the GBR using CRW´s near real-time satellite-based heat stress products. They described an unprecedented event that hit the historical records in the onset of bleaching conditions and its magnitude.*

*Taking as a reference the Stress Level categories, used in Coral Reef Watch (CRW), they forecast a mass coral bleaching in the next season. While the description is detailed, the study is limited by focusing only on thermal exposure; NOAA’s DHW tends to overpredict events of bleaching.*

**Response 1:**

 The present study is a description of unprecedented early-summer sea surface temperature (SST) conditions, and a description of forecast SST conditions. Our aim was to alert managers, scientists, and other stakeholders, who work on the Great Barrier Reef (GBR) to these conditions and forecasts. While a critique of the Degree Heating Week (DHW) algorithm in general may not be unwarranted, this manuscript is not a justification of, or investigation into the DHW algorithm. Here, we focus firstly on the comparison of satellite SST among years from 1985-2021, then look into the heat stress conditions by simply utilising the DHW metric as a tool to describe and compare the observed and forecasted heat stress accumulation on the GBR. For a location with known overprediction by DHW, the comparison of DHW values with previous years is still valid in describing unprecedented SST conditions. This article describes recorded unprecedented early-summer conditions, and alerts readers to the forecast of anomalous heat stress accumulation on the GBR for the 2021-2022 summer season.   

***Comment 2:*****

*Considering other factors is becoming necessary because at a regional and local scale there are several factors that could change the predictions and avoid overpredictions (error type I). For example, turbidity can exacerbate (if linked to water quality, e.g., Wooldridge 2020* *^1^* *) or mitigate the effects (if linked to the reduction of light stress, e.g., Sully and Van Woesik 2020* *^2^* *, Gonzalez-Espinosa and Donner 2021* *^3^* *). However, even though there is evidence that the susceptibility of coral communities to mass bleaching from thermal stress is constrained to the local biological and environmental contexts, and multivariate prediction methods that incorporate other environmental variables (e.g., solar radiation, or light attenuation) and other temperature metrics (e.g. heating rate, high-frequency variability, bimodality) have suggested an improvement in predicting the spatial variability in bleaching intensity for individual events (Maina et al., 2008* *^4^* *; McClanahan et al., 2019* *^5^* *; Safaie et al., 2018* *^6^* *; Sully & van Woesik, 2020* *^2^* *), the algorithms used to predict bleaching (as an operational tool) remain focused only on temperature.*

**Response 2:**

 While several factors can be responsible for coral bleaching at regional and local scales, there is a strong relationship between large-scale bleaching (i.e. mass coral bleaching) and thermal stress accumulation. The focus of this study is strictly on the description of large-scale accumulated heat stress, leading to mass coral bleaching, not on small-scale or local bleaching events.

 In this study, we found that the NOAA Coral Reef Watch (CRW) Four-Month Coral Bleaching Heat Stress Outlook product had forecast heat stress accumulation for 45% of the 5,274 GBR pixels (0.05 x 0.05 degrees) to match or exceed that which has been demonstrated to result in mass bleaching events on the GBR in the past (since 1985). We therefore found it appropriate to alert managers, scientists and other stakeholders working on the GBR that these thermal conditions were forecasted, and that if the forecast conditions were correct, there would be a high probability of a mass bleaching event taking place in these areas. The CRW Outlook product forecast described in this manuscript provides a likelihood of thermal stress accumulation levels, categorized in relation to potential bleaching.

 As you mention, the algorithms currently available to predict mass coral bleaching remain focused only on temperature. The aim of this study was not to improve upon those algorithms, nor to create a new predictive tool. Our goal was to provide a timely report of the unprecedented early-summer SST conditions as well as a warning of SST conditions forecast for the GBR, in the context of a potential mass bleaching event. However, it is worth highlighting that the 2021-2022 summer season has since passed, and CRW’s 21 December 2021 forecast of accumulated heat stress levels has been confirmed by subsequent near real-time satellite SST data to be largely accurate. Further, the effects of this heat stress have been confirmed, via aerial and in-water surveys, to have resulted in a mass coral bleaching event that encompassed the far northern and central GBR.

***Comment 3:***

*Finally, I would be cautious with the assumption that mass bleaching “has only been linked to the stress of excess sea surface temperatures” because even though the elevated temperature is often reported as the primary cause of observed mass coral bleaching, it is the interaction between temperature and other environmental variables that modulate bleaching responses. A quick exploratory analysis of other environmental variables (e.g., description or time series of turbidity, cloudiness, etc) could be included in the discussion.*

*On the other hand, since this study is focused on describing the thermal exposure using a single metric approach then it successfully reports the unprecedented conditions in the historical record.*

**Response 3:**

 Thank you for this comment. We agree that the quoted statement could have been more carefully phrased. We have edited this section of the text to now read:

 “Mass coral bleaching (large-scale bleaching of an entire reef system or geographic realm) has, with few exceptions, always been linked to the stress of excess sea surface temperatures (SST), and this is expected to happen when heat stress in a region exceeds a certain intensity or duration (Glynn, 1984; Hoegh-Guldberg, 1999; Skirving *et al*., 2019).”

 We appreciate your suggestion of an exploratory analysis of other environmental variables influencing coral bleaching, however, this is beyond the scope of our study. This study, as you acknowledge, is focused on describing a single metric (thermal stress accumulation) in relation to the GBR for the 2021-2022 summer season.

# Peer review: Reviewer response for version 1

**DOI:** 10.5256/f1000research.120142.r129136

**Published:** 2022-04-14

**Authors:** Timothy R. McClanahan

**Affiliations:** 1 Global Marine Programs, Wildlife Conservation Society, Bronx, NY, USA

**Coral bleaching is a biological response poorly predicted by excess heat**

 The paper describes the excess temperature or Degree-heating Weeks (DHW) in the Great Barrier Reef (GBR) up to December 2021. They show that excess heat is above historical records, which is increasingly becoming a common report as climate change warms the oceans. The authors predict a mass bleaching in subsequent months. The paper is descriptive and uses NOAA predictions based on thresholds that have not been modified since established in 2012, despite a literature providing ways to improve the algorithms (van Hooidonk et al. 2013; DeCarlo 2020). NOAA provides these predictions on a global basis and here they present it for the GBR during an unusual ocean state, or La Nina year.

 Coral bleaching is a biological response and therefore there are several problems with using global algorithmic predictions based on thermal exposure alone. Corals constitute many species and associated life histories that have evolved in different thermal and stress environments. Responses to excess heat is complex and seems to be changing over time and greatly influenced by geography and other environmental factors (Sully et al. 2020; McClanahan et al. 2019, 2020a,b). Therefore, the DHW for predicting bleaching has high variance and low skill or predictive ability (van Hooidonk and Huber 2009; DeCarlo 2020). Therefore, many studies in this same region find various responses and not high bleaching when thresholds have been passed (Kim et al. 2019; DeCarlo and Harrison 2020). Other examples are that an early peak temperature can prompt an acclimation response that can prevent or reduce bleaching (Ainsworth et al. 2016). Bleaching is typically a late-summer response, and an early peak is not always an indicator of late-season bleaching. Other studies show that other forces like light and nutrients can modify the response. Some differences have been attributed to cloudiness and light, which may have been more important modifier than is broadly recognized in the past events (McGowan and Theobald 2017). Other studies in this region suggest that high nutrients due to ocean currents and upwelling will increase bleaching responses (DeCarlo et al. 2019). Finally, GBR corals are displaying decreasing sensitivity to thermal stress, which is likely to result in more false positives in prediction looking into the future (Hughes et al. 2019).

 In short, there are many unknowns surrounding the coral bleaching phenomenon, which makes it quite difficult to predict bleaching even from early summer warming. Even some of the best studies of predictive skill suggest the highest values rarely exceed 35% for DHW due to both many false negatives and positives (Logan et al. 2012). Moreover, the number of false alarms appears to be increasing since 2000, which is likely due to the original algorithm weakening as corals change and adapt to thermal exposures (DeCarlo 2020). When predicting bleaching for such a large area as the GBR, the skill increases, but this is not surprising and tells us less about bleaching reoccurrence, but rather that there is high spatial variability likely to capture bleaching as the area of prediction increases. The frequency of bleaching at constant spatial cell sizes is not increasing over time (Skirving et al. 2019). The frequency may appear to be increasing if increasing numbers of observers are recording bleaching in large areas, such as national boundaries (Hughes et al. 2018). In sum, there is no discrete or generic global threshold for coral bleaching or mortality but rather high spatial and changing variability. The duration and spatial extent of bleaching is what is increasing and likely leading to more phenomena known as heat waves (Skirving et al. 2019).

 This Coral Reef Watch excess heat has global coverage that would encompass many areas influenced by the La Nina, not just the GBR. The key issue that needs to be addressed is the excess heat on western ocean boundaries during a protracted inter-annual La Nina.  How has the whole Pacific responded and where is the excess heat in this year relative to other La Nina years? I see this large-scale geography as the important aspect of this period in terms of novel responses and predictions. Are there some other processes influencing this La Nina relative to the previous years?  This sort of detective work would be highly appreciated and continue to add to the context and nuances around climate change and inter-annual ocean oscillations.

 The authors need to place their work in the context of the findings and ocean states above. The current version lacks the context and nuance demanded by a critical reading of the scientific literature on bleaching. Catastrophic framing should be avoided, especially without acknowledging the full context or other possible factors or interpretations. The science of bleaching has moved on considerably since some of these early warning DHW algorithms were developed (DeCarlo 2020). The algorithms need to change to accommodate this increase in knowledge and to include other variables that are available from satellite data (Gonzalez‐Espinosa and Donner 2021; McClanahan and Azali 2021).

 In short, this is a report about excess temperature and not the biological response of corals. Coral bleaching is as much about coral sensitivity as it is about excess heat, and this requires more than satellite data to evaluate.

Is the work clearly and accurately presented and does it cite the current literature?

No

If applicable, is the statistical analysis and its interpretation appropriate?

No

Are all the source data underlying the results available to ensure full reproducibility?

Partly

Is the study design appropriate and is the work technically sound?

Partly

Are the conclusions drawn adequately supported by the results?

Partly

Are sufficient details of methods and analysis provided to allow replication by others?

Yes

Reviewer Expertise:

Coral reef ecology

I confirm that I have read this submission and believe that I have an appropriate level of expertise to confirm that it is of an acceptable scientific standard, however I have significant reservations, as outlined above.

## References

[1] : Climate change disables coral bleaching protection on the Great Barrier Reef. *Science* .2016;352(6283) : 10.1126/science.aac7125 338-42 10.1126/science.aac7125 27081069

[2] : An enigmatic decoupling between heat stress and coral bleaching on the Great Barrier Reef. *PeerJ* .2019;7: 10.7717/peerj.7473 e7473 10.7717/peerj.7473 31423360PMC6694787

[3] : Nutrient-supplying ocean currents modulate coral bleaching susceptibility. *Sci Adv* .6(34) : 10.1126/sciadv.abc5493 10.1126/sciadv.abc5493 32937375PMC7442482

[4] : Treating coral bleaching as weather: a framework to validate and optimize prediction skill. *PeerJ* .2020;8: 10.7717/peerj.9449 e9449 10.7717/peerj.9449 32685288PMC7337031

[5] : Cloudiness reduces the bleaching response of coral reefs exposed to heat stress. *Glob Chang Biol* .27(15) : 10.1111/gcb.15676 3474-3486 10.1111/gcb.15676 33964101

[6] : Quantifying the quality of coral bleaching predictions. *Coral Reefs* .2009;28(3) : 10.1007/s00338-009-0502-z 579-587 10.1007/s00338-009-0502-z

[7] : Temporary refugia for coral reefs in a warming world. *Nature Climate Change* .2013;3(5) : 10.1038/nclimate1829 508-511 10.1038/nclimate1829

[8] : Global warming and recurrent mass bleaching of corals. *Nature* .2017;543(7645) : 10.1038/nature21707 373-377 10.1038/nature21707 28300113

[9] : Spatial and temporal patterns of mass bleaching of corals in the Anthropocene. *Science* .2018;359(6371) : 10.1126/science.aan8048 80-83 10.1126/science.aan8048 29302011

[10] : Ecological memory modifies the cumulative impact of recurrent climate extremes. *Nature Climate Change* .2019;9(1) : 10.1038/s41558-018-0351-2 40-43 10.1038/s41558-018-0351-2

[11] : Refugia under threat: Mass bleaching of coral assemblages in high-latitude eastern Australia. *Glob Chang Biol* .25(11) : 10.1111/gcb.14772 3918-3931 10.1111/gcb.14772 31472029

[12] : Temperature patterns and mechanisms influencing coral bleaching during the 2016 El Niño. *Nature Climate Change* .2019;9(11) : 10.1038/s41558-019-0576-8 845-851 10.1038/s41558-019-0576-8

[13] : Large geographic variability in the resistance of corals to thermal stress. *Global Ecology and Biogeography* .2020;29(12) : 10.1111/geb.13191 2229-2247 10.1111/geb.13191

[14] : Highly variable taxa-specific coral bleaching responses to thermal stresses. *Marine Ecology Progress Series* .2020;648: 10.3354/meps13402 135-151 10.3354/meps13402

[15] : Environmental Variability and Threshold Model’s Predictions for Coral Reefs. *Frontiers in Marine Science* .2021;8: 10.3389/fmars.2021.778121 10.3389/fmars.2021.778121

[16] : ENSO Weather and Coral Bleaching on the Great Barrier Reef, Australia. *Geophysical Research Letters* .2017;44(20) : 10.1002/2017GL074877 10,601-10,607 10.1002/2017GL074877

[17] : The relentless march of mass coral bleaching: a global perspective of changing heat stress. *Coral Reefs* .2019;38(4) : 10.1007/s00338-019-01799-4 547-557 10.1007/s00338-019-01799-4

[18] : A global analysis of coral bleaching over the past two decades. *Nature Communications* .2019;10(1) : 10.1038/s41467-019-09238-2 10.1038/s41467-019-09238-2 PMC642703730894534

# Response

**Published:** 2022-05-17

**Authors:** Blake Spady

**Affiliations:** National Oceanic and Atmospheric Administration, Australia

We would like to express our gratitude for the valuable and thoughtful feedback provided by the reviewer. Please find our response to each comment below.

***Comment 1:***

***Coral bleaching is a biological response poorly predicted by excess heat***

*The paper describes the excess temperature or Degree-heating Weeks (DHW) in the Great Barrier Reef (GBR) up to December 2021. They show that excess heat is above historical records, which is increasingly becoming a common report as climate change warms the oceans. The authors predict a mass bleaching in subsequent months. The paper is descriptive and uses NOAA predictions based on thresholds that have not been modified since established in 2012, despite a literature providing ways to improve the algorithms (van Hooidonk et al. 2013; DeCarlo 2020). NOAA provides these predictions on a global basis and here they present it for the GBR during an unusual ocean state, or La Nina year.*

**Response 1:**

 It is correct that coral bleaching is a biological response, and several factors aside from thermal exposure can be responsible for this process. However, in this paper we are focusing only on mass coral bleaching events, which have a much stronger relationship to thermal stress than bleaching more generally. Your statement that this paper is descriptive is exactly correct; the purpose of this paper was to describe the unprecedented early-summer anomalous sea surface temperature (SST) conditions on the Great Barrier Reef (GBR). Further, we sought to alert managers and scientists on the GBR to these conditions as well as to the forecasted SST conditions for the remainder of the summer season. While it is valid to critique the Degree Heating Week (DHW) algorithm in general, this paper is not a justification of, or investigation into that algorithm. The DHW algorithm utilised in this paper is a widely used tool which quantifies SST heat accumulation over time. Here, we simply use that metric to describe the conditions observed and those forecasted for the remainder of the season. It may also be worth noting that the forecast made on 21 December 2021 of bleaching-level heat stress described within this paper has since been supported by subsequent measurements and observations. The levels of heat stress accumulation predicted through to 13 February 2022 has been confirmed to be largely accurate by the near real-time satellite data. Further, the connection of this heat stress accumulation to an outbreak of mass bleaching has been supported by subsequent field observations. In March 2022, large sections of the far northern GBR and central GBR, the same general areas described here, were observed and described by several third-party sources to have undergone a significant mass bleaching event.  

***Comment 2:***

*Coral bleaching is a biological response and therefore there are several problems with using global algorithmic predictions based on thermal exposure alone. Corals constitute many species and associated life histories that have evolved in different thermal and stress environments. Responses to excess heat is complex and seems to be changing over time and greatly influenced by geography and other environmental factors (Sully et al. 2020; McClanahan et al. 2019, 2020a,b). Therefore, the DHW for predicting bleaching has high variance and low skill or predictive ability (van Hooidonk and Huber 2009; DeCarlo 2020). Therefore, many studies in this same region find various responses and not high bleaching when thresholds have been passed (Kim et al. 2019; DeCarlo and Harrison 2020). Other examples are that an early peak temperature can prompt an acclimation response that can prevent or reduce bleaching (Ainsworth et al. 2016). Bleaching is typically a late-summer response, and an early peak is not always an indicator of late-season bleaching. Other studies show that other forces like light and nutrients can modify the response. Some differences have been attributed to cloudiness and light, which may have been more important modifier than is broadly recognized in the past events (McGowan and Theobald 2017). Other studies in this region suggest that high nutrients due to ocean currents and upwelling will increase bleaching responses (DeCarlo et al. 2019). Finally, GBR corals are displaying decreasing sensitivity to thermal stress, which is likely to result in more false positives in prediction looking into the future (Hughes et al. 2019).*

**Response 2:**

 We concede that the biological response of coral bleaching is a complicated multi-variate process and we don’t claim here that the DHW algorithm alone has the capacity to predict bleaching in all forms, uniformly across all regions, or equally effectively at all spatial scales. However, at large scales (i.e. mass bleaching events), the DHW algorithm used here has been effective at demonstrating a strong correlation between heat-stress accumulation and significant coral bleaching. Considering that correlation, the aim of this paper was again to alert scientists and managers to an unprecedented thermal anomaly observed in the early-summer 2021-2022 on the GBR, and to raise awareness of the potential of a large-scale bleaching event. We agree with your statement of “ *Bleaching is typically a late-summer response, and an early peak is not always an indicator of late-season bleaching.*” It is for that reason that we found it necessary to highlight this event as it was unfolding. At the time of writing this paper we had seen an unprecedented build-up of thermal stress on the GBR, and the forecast at the time did not indicate that this was an early peak, but that heat accumulation would continue to build through January and into February. It was also found that the forecasted sum of this accumulated heat was that which has been shown to result in mass bleaching events on the GBR in the past. While the field observations of mass coral bleaching along the northern half of the GBR in March 2022 cannot confirm that bleaching had occurred prior to February 2022, the data we had in mid-December 2021 indicated that this would be a possibility. Therefore, we sought to alert those working specifically on the GBR of this potential.     

   

***Comment 3:***

*In short, there are many unknowns surrounding the coral bleaching phenomenon, which makes it quite difficult to predict bleaching even from early summer warming. Even some of the best studies of predictive skill suggest the highest values rarely exceed 35% for DHW due to both many false negatives and positives (Logan et al. 2012). Moreover, the number of false alarms appears to be increasing since 2000, which is likely due to the original algorithm weakening as corals change and adapt to thermal exposures (DeCarlo 2020). When predicting bleaching for such a large area as the GBR, the skill increases, but this is not surprising and tells us less about bleaching reoccurrence, but rather that there is high spatial variability likely to capture bleaching as the area of prediction increases. The frequency of bleaching at constant spatial cell sizes is not increasing over time (Skirving et al. 2019). The frequency may appear to be increasing if increasing numbers of observers are recording bleaching in large areas, such as national boundaries (Hughes et al. 2018). In sum, there is no discrete or generic global threshold for coral bleaching or mortality but rather high spatial and changing variability. The duration and spatial extent of bleaching is what is increasing and likely leading to more phenomena known as heat waves (Skirving et al. 2019).*

**Response 3:**

 We must stress that the predictions made within this paper were more specifically of the Alert Levels detailed in **Table 2**, which are categorisations of thermal stress related to potential bleaching. Further, we stress that the early-summer heat stress detailed is not what we used to predict this accumulated heat stress. The predictions of heat stress made in this paper were derived from the NOAA Coral Reef Watch modelled weekly global Four-Month Coral Bleaching Heat Stress Outlook product (CRW Outlook product). The CRW Outlook product uses SST forecasts generated by the NOAA/National Weather Service/National Centers for Environmental Prediction’s Climate Forecast System Version 2 (CFSv2) to generate weekly predictions of the likelihood of heat stress within the Alert Levels (referenced above) up to 20 weeks into the future. The intent of this paper was not to use the early-summer heat stress as a predictive indicator of conditions over the following months, but to describe both the unprecedented early-summer conditions as well as to describe the forecast of accumulated heat stress for the upcoming summer months. As you mention here, and as we allude to in our response to **Comment 2**, the predictive skill of the DHW algorithm increases at larger spatial scales. For that reason, we find the use of the DHW algorithm justified and appropriate in this context. The forecast of accumulated heat stress described here covered 45% of the 5,274 GBR pixels (0.05 x 0.05 degrees), or approximately 59,332 km ^2^. This gave us relatively high confidence that, if the forecast was correct, significant portions of this large area would experience heat stress related bleaching. We fully agree that there is no discrete or generic global threshold for coral bleaching, however, we also know that thermal stress is almost always related to mass coral bleaching and that once a certain level of thermal stress is exceeded, a bleaching event is likely.

**Comment 4:**

*This Coral Reef Watch excess heat has global coverage that would encompass many areas influenced by the La Nina, not just the GBR. The key issue that needs to be addressed is the excess heat on western ocean boundaries during a protracted inter-annual La Nina.  How has the whole Pacific responded and where is the excess heat in this year relative to other La Nina years? I see this large-scale geography as the important aspect of this period in terms of novel responses and predictions. Are there some other processes influencing this La Nina relative to the previous years?  This sort of detective work would be highly appreciated and continue to add to the context and nuances around climate change and inter-annual ocean oscillations.*

**Response 4:**

 Thank you for this comment. We agree that this is indeed a key issue and an interesting academic pursuit. The scope of this paper, as you mention in **Comment 1**, is descriptive in nature. Our aim here was to inform managers and scientists who work on the GBR of the facts revealed by the data, and not to investigate the precise causes and effects of these observations. We encourage readers of this manuscript to pursue the answers to these questions, as it is beyond the scope of this study.   

***Comment 5:***

*The authors need to place their work in the context of the findings and ocean states above. The current version lacks the context and nuance demanded by a critical reading of the scientific literature on bleaching. Catastrophic framing should be avoided, especially without acknowledging the full context or other possible factors or interpretations. The science of bleaching has moved on considerably since some of these early warning DHW algorithms were developed (DeCarlo 2020). The algorithms need to change to accommodate this increase in knowledge and to include other variables that are available from satellite data (Gonzalez‐Espinosa and Donner 2021; McClanahan and Azali 2021).*

*In short, this is a report about excess temperature and not the biological response of corals. Coral bleaching is as much about coral sensitivity as it is about excess heat, and this requires more than satellite data to evaluate.*

**Response 5:**

 Thank you. As mentioned in our response to **Comment 4**, this paper is purely descriptive. Therefore, it is beyond the scope of this study to relate the findings from the thermal event described here to the ocean conditions and ocean states elsewhere in the Pacific. We do agree that catastrophic framing should be avoided. However, we do not feel that our framing of the predictions of bleaching-level heat stress is “catastrophic”. We make no predictions or mention of subsequent coral mortality, recovery, or of the ecological impacts of the predicted potential bleaching event. Further, we must reiterate that while we framed our predictions as being “potential” within the paper, it is important to note that the 2021-2022 summer season on the GBR has since passed and our predictions have been confirmed to be largely accurate based on both satellite data and in-water and aerial surveys of the GBR. Precisely how accurate these predictions were cannot yet be determined, but it is clear that a mass coral bleaching event took place in the 2021-2022 summer season within the areas that we forecasted bleaching-level heat stress on 21 December 2021. We can appreciate your critiques of the DHW algorithm, but we must also stress that this paper is not an investigation into, nor a justification of that algorithm. This paper was only ever intended to be a timely report of excess SST on the GBR, as well as a modelled outlook of forecasted SST conditions in the context of coral stress. As such we merely use the DHW algorithm as a tool to describe these conditions and compare historical conditions among years.
